# Maximizing the Accuracy of Continuous Quantification Measures Using Discrete PackTest Products with Deep Learning and Pseudocolor Imaging

**DOI:** 10.1155/2019/1685382

**Published:** 2019-04-09

**Authors:** Ryoichi Doi

**Affiliations:** Faculty of Social-Human Environmentology, Daito Bunka University, 1-9-1 Takashimadaira, Itabashi-ku, Tokyo 175-8571, Japan

## Abstract

Using the standard colors provided in the instructions, PackTest products can approximate and quickly estimate the chemical characteristics of liquid samples. The combination of PackTest products and deep learning was examined for its accuracy and precision in quantifying chemical oxygen demand, ammonium ion, and phosphate ion using a pseudocolor imaging method. Each PackTest product underwent reactions with standard solutions. The generated color was scanner-read. From the color image, ten grayscale images representing the intensity values of red, green, blue, cyan, magenta, yellow, key black, and *L*^*∗*^, and the values of *a*^*∗*^ and *b*^*∗*^ were generated. Using the grayscale images representing the red, green, and blue intensity values, 73 other grayscale images were generated. The grayscale intensity values were used to prepare datasets for the ten and 83 (=10 + 73) images. For both datasets, chemical oxygen demand quantification was successful, resulting in values of normalized mean absolute error of less than 0.4% and coefficients of determination that were greater than 0.9996. However, the quantification of ammonium and phosphate ions commonly provided false positive results for the standard solution that contained no ammonium ion/phosphate ion. For ammonium ion, multiple regression markedly improved the accuracy using the pseudocolor method. Phosphate ion quantification was also improved by avoiding the use of an estimated value for the reference solution that contained no phosphate ion. Real details of the measurements and the perspectives were discussed.

## 1. Introduction

PackTest products, like test strips, qualitatively indicate chemical characteristics of liquid samples. PackTest products are cost-effective, convenient, quick, easy to use, safe, and have minimal waste and thus are highly feasible to use. There are some ten PackTest products (http://kyoritsu-lab.co.jp/english/). Each product contains dry reagents in a handy, flexible, and elastic semitransparent plastic pack. By pressing the pack to purge the air inside and submerging the pack edge in a liquid sample, the plastic pack takes in the sample liquid from a small hole at the edge of the pack. The reagents react with the chemical to be detected. The reaction generates visible coloration of the entire reaction system in the plastic pack [1]. Thus, the user can visually observe if there is a particular chemical in the liquid sample. The products were originally designed for quick and approximate detection of chemicals, and thus, they are considered to be qualitative or semiquantitative analytical tools. For qualitative or semiquantitative detection, users refer to a series of standard colors that show the approximate concentration of the chemical in question, which generates a color after the chemical reaction. Because of the feasibility, PackTest products may be widely used in various places including secondary schools [2] and field sites with environmental issues [3].

By referring to standard colors, PackTest users find the closest value that is represented by a standard color. For this reason, PackTest was originally a tool with discrete measures for the chemical characteristics. However, recent studies indicated the possibility that the PackTest products can be tools for accurately and quantitatively determining the chemical characteristics of liquid samples [4]. These tools can be used with continuous measures provided by computation such as regression. The continuous measures can solve the difficulty in determining the closest value when a sample color nears the midpoint between two standard colors [5]. Another reason that continuous measures are preferred is that accurate quantitative determination of chemical characteristics is more advantageous than qualitative detection. An example is the critical effects of blood biochemical characteristics at low levels [6]. Improving the accuracy of PackTest products is expected to enable detection of subtle but significant signals [7]. Previous studies aimed to enhance the accuracy of the feasible tools such as test strips by determining the regression models that describe the chemical characteristics [8]. In addition to the regression techniques, deep learning tools are now commonly available.

Deep learning is especially advantageous for analyzing images. Deep learning was used to explore relationships between health and information that was derived from medical images [9]. Besides its application to image analyses, deep learning was expected to extract the patterns of color changes in the PackTest reaction mixture as responses to changes in the level of the chemical characteristics in question. Deep learning evolved from artificial neural network that was well established and used for quantitative determination of chemical characteristics [10]. Deep learning is relatively more tolerant to some limitations, such as overtraining, which more significantly affect artificial neural networks. Recently, freeware for deep learning was launched. To the best of my knowledge, few studies have involved deep learning for quantification of chemical characteristics.

Based on the above background, this study was conducted to apply deep learning to quantify chemical characteristics of liquid samples. These characteristics were chemical oxygen demand, ammonium ion concentration, and phosphate ion concentration. Standard solutions with multiple levels of the chemical characteristics were prepared. Coloration was generated by introducing the standard solutions to the PackTest products. The colored pack was optically scanned. The color image was used as the starting material for 83 grayscale images to enhance the information [4]. The grayscale intensity values for selected pixels that indicate the chemical characteristics were read and used for deep learning. Validating the training results revealed that some chemical characteristics were very accurately quantified. However, for some chemical characteristics, the training was found to have problems. These problems were, however, avoided when complementally techniques were used.

## 2. Materials and Methods

### 2.1. PackTest Products

PackTest products for determining chemical oxygen demand, ammonium (NH_4_^+^) ion, and phosphate (PO_4_^3−^) ion (Kyoritsu Chemical-Check Lab, Corp., Tokyo, Japan) were purchased. PackTest is a series of products for determining the chemical characteristics of liquids (http://kyoritsu-lab.co.jp/english/). The product is a handy and flexible plastic pack that has a hole at the edge. The plastic pack contains a set of reagents that react with the chemical (characteristic) to be measured colorimetrically. After purging the air inside the pack using the hole, the pack edge is submerged in the sample liquid and the sample enters the pack through the hole. Then, the chemical characteristic is approximately determined by referring to standard colors in the instructions that come with the pack. Chemical oxygen demand is detected by applying the principle of alkaline oxidation with potassium permanganate [11]. NH_4_^+^ and PO_4_^3−^ are detected using an indophenol reaction [12] and a molybdenum blue reaction [13], respectively.

### 2.2. Standard Solutions and Reaction

Glucose solutions, the standard solutions for chemical oxygen demand, were prepared by dissolving glucose in distilled water. The concentrations were 1.46, 2.93, 5.86, 11.7, 23.4, 46.6, and 93.8 mg·glucose·L^−1^. These glucose concentrations are 1.56, 3.13, 6.25, 12.5, 25, 50, and 100 mg·chemical·oxygen·demand·L^−1^. Ammonium standard solutions were prepared by dissolving ammonium chloride in distilled water. The concentrations were 35, 69, 139, 278, and 556 *µ*M. Phosphate ion standard solutions were prepared by dissolving sodium dihydrogen phosphate dihydrate in distilled water at 0, 13, 26, 53, and 105 *µ*M.

The edge of a pack for chemical oxygen demand was submerged in the standard glucose solution. The standard solution was introduced into the pack from a small hole at the edge of the pack until it contained solution to a depth of 3.5 cm. Then, the standard solution in the pack was gently stirred by turning the pack, according to the manufacturer's instructions. The reaction took 5 minutes at room temperature (approximately 23°C). Five pack replications were used for each glucose concentration.

Similarly, the phosphate ion (PO_4_^3−^) standard solution was introduced into the pack for phosphate ion detection. The phosphate ion kit contained a small plastic container to accurately take in 1.5 mL of liquid sample into the pack. The reaction took 1 minute at room temperature. The ammonium chloride standard solution was introduced into the pack for the ammonium ion (NH_4_^+^) detection at 1.5 mL·pack^−1^ in a similar manner. The reaction took 5 minutes at room temperature. Three pack replications were used for each NH_4_^+^ or PO_4_^3−^ concentration.

### 2.3. Image Acquisition and Processing

The coloration was read using an Epson GT-S 650 optical scanner (Seiko Epson Corp., Suwa, Japan) at 300 dots per inch in the professional mode. The other settings of the scanner were set at the default. The scanner was placed vertically, and the colored pack was attached at approximately the center of the scanner's bed glass using scotch tape to prevent the solution from leaking out. The scanner lid, with a white plastic pad on the inside, was loosely closed. An image of the colored pack was acquired in the dark. The image was saved as a JPG file and then converted to a tag image file format file in the red-green-blue (RGB) mode with the sRGB color space.

**Figure 1:**
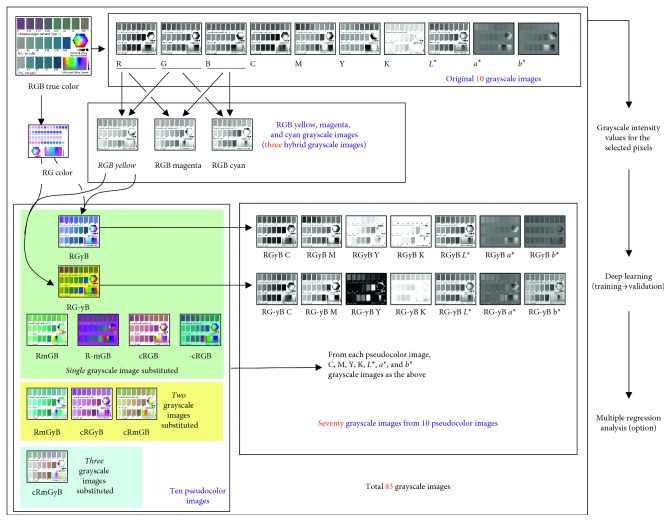
Pseudocolor imaging and data-processing procedures employed in this study. Details of the RGB true-color image are shown in Figure 3. The hexagon and the rectangle at the bottom of the image are Microsoft standard colors and gamut, respectively.

Details of the image processing methods are described elsewhere [4, 14]. With Adobe Photoshop CS2 software (Adobe Systems Inc., California, USA), the tag image file format image generated ten grayscale tag image file format images that indicated the grayscale intensity values for the color components of RGB, cyan-magenta-yellow-key black (CMYK), and the International Commission and Illumination's *L*^*∗*^*a*^*∗*^*b*^*∗*^ color models (Figure 1). CMYK images were generated using the International Color Consortium profile of US Web Coated (SWOP) v2 for digital output such as color printing. Hereafter, these ten grayscale images will be called the original ten grayscale images (Figure 1). An RGB yellow hybrid grayscale image was prepared by merging the R and G grayscale images at the same weights [15]. Similarly, RGB cyan and RGB magenta hybrid grayscale images were prepared by merging the G and B grayscale images and the R and B grayscale images, respectively.

To prepare a pseudocolor RGB image, the entire area of a B grayscale image in an RGB color image was substituted by the RGB yellow grayscale image. Hereafter, this image is called the RGyB image (Figure 1). A pseudocolor RG-yB image was prepared by substituting the B grayscale image of the RGB color image with the black-white inverted RGB yellow grayscale image. Similarly, an RmGB pseudocolor image was prepared by placing the RGB magenta grayscale image onto the entire area of a G grayscale image of an RGB image. R-mGB, cRGB, and -cRGB pseudocolor images were also prepared and saved.

Pseudocolor images carrying two or three of the RGB cyan, RGB magenta, and RGB yellow hybrid grayscale images were also prepared (Figure 1). For example, the G and B grayscale images of an RGB image were substituted with the RGB magenta and yellow hybrid grayscale images, respectively. Hereafter, this image is called the RmGyB pseudocolor image. Similarly, cRGyB and cRmGB pseudocolor images were prepared. By substituting the R, G, and B grayscale images of the RGB color image with the RGB cyan, magenta, and yellow hybrid grayscale images, respectively, a cRmGyB pseudocolor image was also prepared.

The ten pseudocolor images were saved as tag image file format files. Each of the pseudocolor images was converted to CMYK and *L*^*∗*^*a*^*∗*^*b*^*∗*^ color images. Next, grayscale images that showed the intensity values of C, M, Y, K, and *L*^*∗*^ and the values of *a*^*∗*^ and *b*^*∗*^ were prepared from each pseudocolor image (Figure 1). Hereafter, the C, M, Y, K, *L*^*∗*^, *a*^*∗*^, and *b*^*∗*^ images are referred to as the RGyB C grayscale image and so on. Thus, 70 grayscale images were added to the original ten grayscale images and the three hybrid grayscale images of RGB yellow, RGB magenta, and RGB cyan. A total of 83 grayscale images were obtained for each chemical characteristic.

The grayscale intensity values for pixels representing the colors for the standard solutions were read by running MultiSpec version 3.4 for Windows (Purdue Research Foundation, Indiana, USA). The intensity values were digital numbers between 0 (complete black) and 255 (complete white). Within a single pack image, ten replication pixels and five other pixels were selected as training and validation pixels, respectively. The pixels were selected in an image area with the least diffuse reflection. The grayscale intensity values for the selected pixels were read for the 83 grayscale images. Grayscale datasets based on the original ten (RGB, CMYK, and *L*^*∗*^*a*^*∗*^*b*^*∗*^) and all 83 images were prepared and compared in terms of accuracy of quantification of the chemical characteristics.

To train deep learning of relationships between coloration and chemical oxygen demand levels, the grayscale intensity values for the ten replication pixels × five packs × eight chemical oxygen demand levels, 400 pixels were used. Similarly, to estimate NH_4_^+^ and PO_4_^3−^ concentrations, the grayscale intensity values for the ten replication pixels × three packs × six (NH_4_^+^) or five (PO_4_^3−^) levels thus 180 (NH_4_^+^) or 150 (PO_4_^3−^) training pixels were used. Besides the training pixels, validation pixels were selected. The numbers of pixels used for the validation were 200 (chemical oxygen demand), 90 (NH_4_^+^), and 75 (PO_4_^3−^).

### 2.4. Deep Learning and Related Techniques

**Figure 2:**
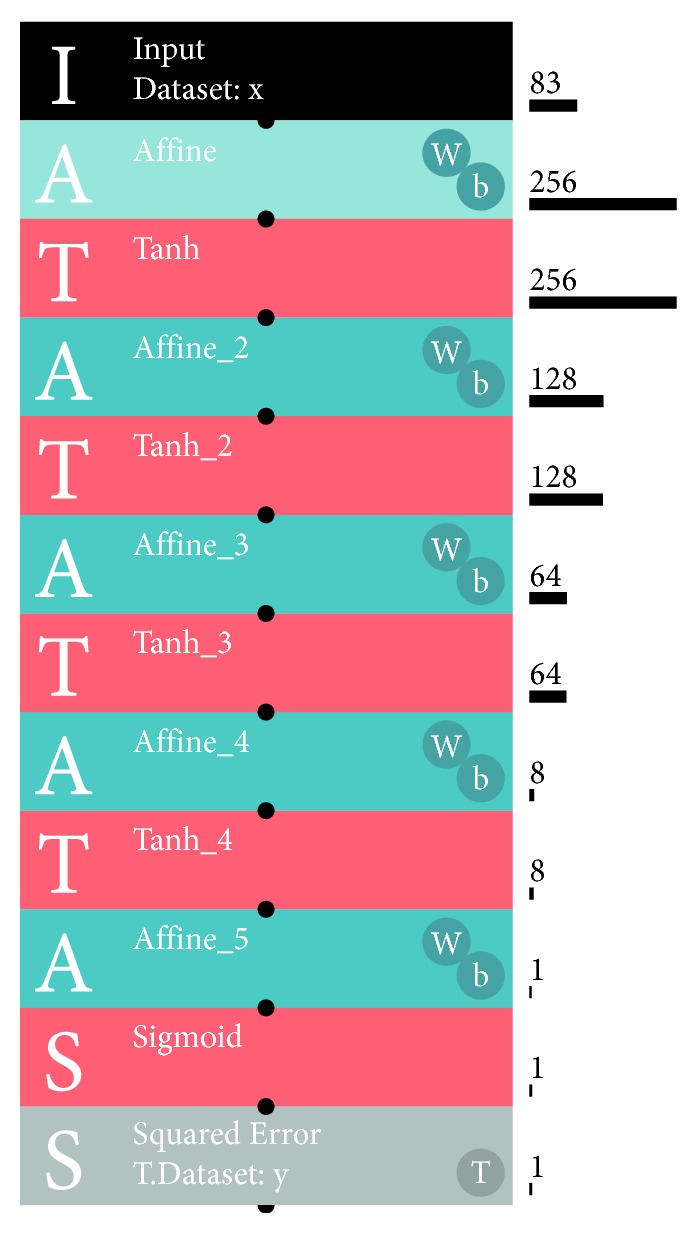
Deep learning architecture used in this study. Affine is a networking structure. Tanh is a process of hyperbolic tangent-conversion of the input. Sigmoid converts a value generated by the upstream processes. Sigmoid results in a sigmoid-converted value between 0 and 1. Squared Error is a process for minimizing errors in the estimation of the value.

Using a SONY neural network console 1.20 (SONY Corp., Tokyo, Japan), deep learning was performed. From the basic series of architecture, 10_deep_mlp.sdcproj was selected. The architecture consisted of five layers (Figure 2). In Figure 2, Affine is a networking structure and Tanh is a process in which hyperbolic tangent-converts a value generated by the upstream processes to provide a converted value between −1 and 1. Sigmoid converts a value generated by the upstream processes to provide a sigmoid-converted value between 0 and 1. The last process of this architecture was BinaryCrossEntropy for binarization of the input data. However, in this study, it was substituted by SquaredError to minimize errors in the estimation of values. Eighty-three and the other values on the right side along the architecture diagram (Figure 2) are the number of values inputted and processed in the layer. When values derived from the ten grayscale images were inputted, the first input number was ten instead of 83. The raw grayscale intensity values were divided by 255 before the values were processed by the architecture, as recommended by the manufacture's manual. For training and validation, ten and five grayscale intensity values were used for each plastic pack, respectively. The default settings were used in the training and validation except that the number of epochs was 3000. By confirming the discrepancy between changes in training and validation errors, overtraining was monitored.

To complement the quantification using the grayscale intensity datasets and deep learning, the author used the statistical software IBM SPSS Statistics V.24.0 (IBM Corp., New York, USA). The multiple regression model that most significantly describes changes in the chemical characteristic was identified using the stepwise method at the default criteria (*p*=0.05 for inclusion and 0.10 for removal). This may generate multiple regression models. However, those that consist of any coefficient with a variance inflation factor of ten or greater were eliminated because the coefficient was unreliable [16].

Through the above processes, the normalized mean absolute error [17] and coefficient of determination (*R*^2^) were obtained as indicators of accuracy and precision, respectively. The normalized mean absolute error was determined as follows:

(1) $$\begin{array}{c} \text{normalized mean absolute error}\left(\text{\%}\right)=100\times \frac{\left(\sum \left|\text{estimated value}-\text{actual value}\right|\right)}{\left(\text{number of used pixels}/\text{range of value}\right)}. \end{array}$$

The values of these statistics were compared among the best regression models for the datasets for the ten and 83 images to investigate the effects of pseudocolor imaging on the precision and accuracy of determining the chemical characteristics.

Another error statistic was used to confirm the reproducibility of the scanner-read color intensity values. The statistic called the coefficient of variation was determined as follows:

(2) $$\begin{array}{c} \text{coefficient of variation}\left(\%\right)=100\times \frac{\text{standard deviation}}{\text{mean}}. \end{array}$$

The reproducibility was evaluated by reading the color intensity values for 30 selected Microsoft Office standard colors (Figure 1) that were printed on white paper. Using the Epson GT‐S 650 scanner, the colors were read eight times, and thus, eight RGB color images were obtained. Coefficients of variation for the intensity values of redness, greenness, and blueness were determined for the 30 standard colors.

To observe the performance of the 83 grayscale images that were used in this study, a color gamut [14] and the standard colors in Figure 1 were used. The color gamut and standard colors were provided by Microsoft Office 2016. The tag image file format image of the gamut and standard colors was used to obtain the colors' profiles by reading the grayscale intensity values for the 83 grayscale images. There were 144 standard colors. In the gamut, 187 pixels were randomly selected. The grayscale intensity values were read for the 331 pixels in the 83 grayscale images. The intensity values for the 331 pixels × 83 images were used for principal component analysis using the IBM SPSS software.

## 3. Results and Discussion

**Figure 3:**
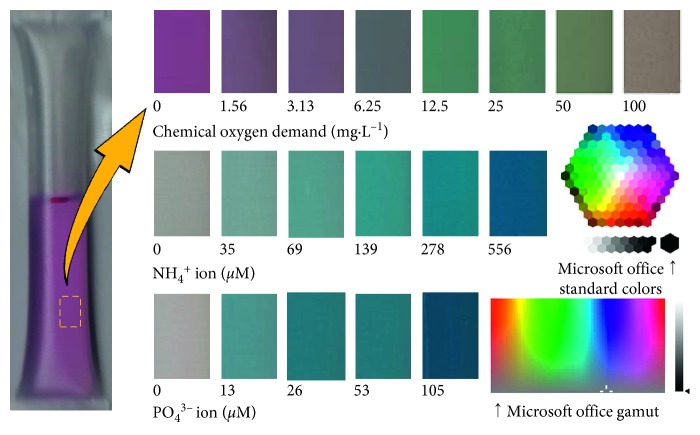
Color development of the PackTest products with standard solutions of chemical oxygen demand, ammonium chloride, or sodium dihydrogen phosphate at different concentrations and incubated at room temperature (approximately 23°C). For each pack, 2400 pixels were copied from the original JPG file obtained using the optical scanner.

**Figure 4:**
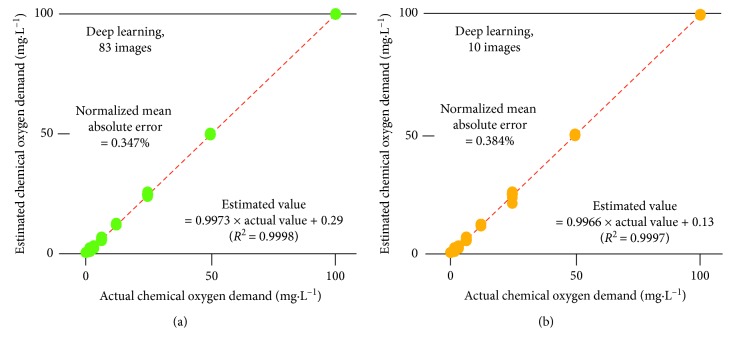
Estimation of chemical oxygen demand by using deep learning based on the 10 and 83 grayscale images derived from the coloration image (Figure 3). The oblique red lines indicate perfect matching between actual and estimated values.

Figure 3 shows color development in the plastic packs. When visually observed, the colors were comparable to those in the instructions provided by the manufacturer, but the images obtained by the scanner were less colorful than those in the instructions. The semitransparency of the plastic material may be at least partially responsible for the relative darkness. Based on the grayscale intensity values derived from the scanner-acquired colors, the chemical oxygen demand was accurately quantified (Figure 4). Training and validation curves overlapped well, and thus, there was no overtraining. The normalized mean absolute errors were 0.384% and 0.347% for the original ten and 83 images' datasets, respectively. The normalized mean absolute error was approximately comparable to the corresponding coefficient of variation [4]. From this viewpoint, the normalized mean absolute error was very small compared with those regarded as acceptable values of 10% in areas of analytical chemistry [18] or 5 to 10% in food chemistry [19].

**Figure 5:**
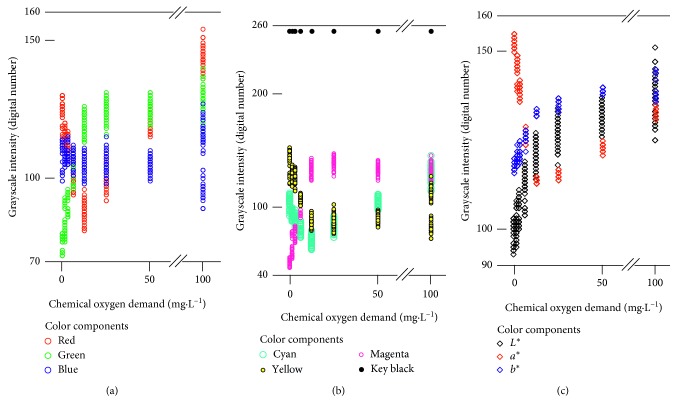
Nonlinear and nonmonotonic relationships between chemical oxygen demand and the grayscale intensity values for the selected training pixels in the original 10 grayscale images (red, green, blue, cyan, magenta, yellow, key black, *L*^*∗*^, *a*^*∗*^, and *b*^*∗*^ images) derived from the color images acquired by the optical scanner.

The chemical oxygen demand has been determined using various methods. Some researchers developed sophisticated methods such as a flow chemiluminescence method [11]. Less complicated methods are colorimetry [20] and titration [21], which are more complicated and time-consuming than the current method. Thus, the PackTest product for chemical oxygen demand could be an advantageous alternative for quantitative determination of the chemical oxygen demand. However, based on the coloration, it should have been difficult to use this tool to its fullest because changes in color are complicated.  Figure 5 demonstrates this complexity. When the intensity values for the pixels in the original ten grayscale images were investigated, no monotonic increase/decrease in chemical oxygen demand was recognized. These complex patterns of color change as responses to changes in chemical oxygen demand of samples disabled the simple empirical description of chemical oxygen demand using a single color component of RGB, CMTK, and *L*^*∗*^*a*^*∗*^*b*^*∗*^ color models.

Another difficulty was suggested. The intensity values for each color component at a single chemical oxygen demand level showed errors (Figure 5). For example, the grayscale intensity values for the pixels in the B grayscale image ranged from less than 90 to 120. The scanner was investigated for its role in this difficulty. The minimum grayscale intensity values for the selected 30 Microsoft standard colors were 47 (redness), 32 (greenness), and 28 (blueness). The maximum values were 253 (redness), 243 (greenness), and 244 (blueness). The coefficient of variation (*N*=8 scanning trials) ranged between 0.2% and 9.1% (redness), 0.3% and 9.0% (greenness), and 0.4% and 15.1% (blueness). The mean coefficient of variation (*N*=30 colors) was 3.0% (redness), 3.9% (greenness), and 3.3% (blueness).

Therefore, the scanner was demonstrated to have good reproducibility. A more likely source of the errors was an inconsistency in attaching the pack onto the scanner's glass bed. The pack was attached each time as consistently as possible. However, in the interface between the glass bed and the pack's surface, slight differences should have occurred. The differences among the replication color readings were thought to be responsible for the errors indicated by the raw grayscale intensity values in Figure 5. However, the deep learning architecture completely eliminated the errors in the training processes. According to Figures 3 and 4, a chemical oxygen demand value of 4 mg·L^−1^ or lower is detectable by combining the PackTest product and deep learning. A chemical oxygen demand of 4 mg·L^−1^ or lower is favorable for a fresh water trout subspecies of *Oncorhynchus masou* [22], which is an important fish species ecologically and as a foodstuff. The current combination of PackTest and deep learning enabled the accurate detection of low levels of chemical oxygen demand for water samples that were polluted at levels that were marginally critical for *O. masou*.

**Figure 6:**
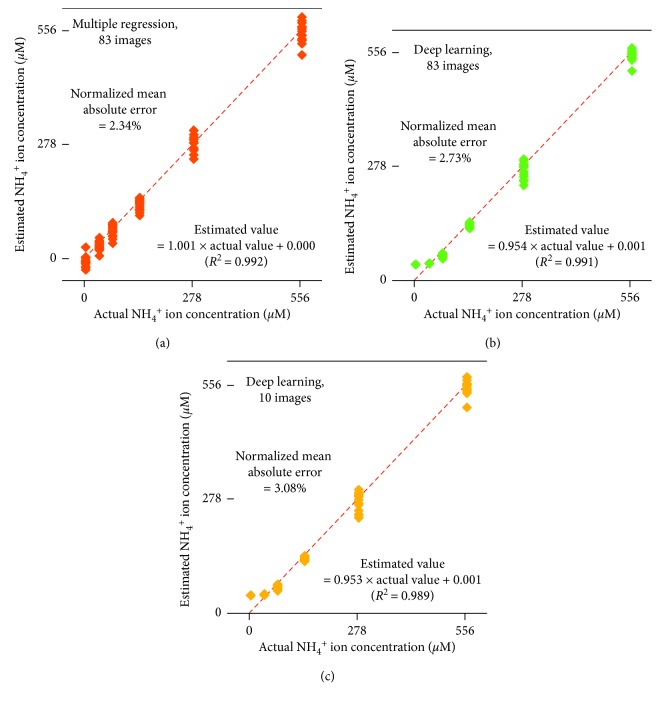
Estimation of ammonium ion (NH_4_^+^) concentration by using deep learning and multiple regression based on the 10 and 83 grayscale images derived from the coloration image (Figure 3). The oblique red lines indicate perfect matching between actual and estimated values.

**Figure 7:**
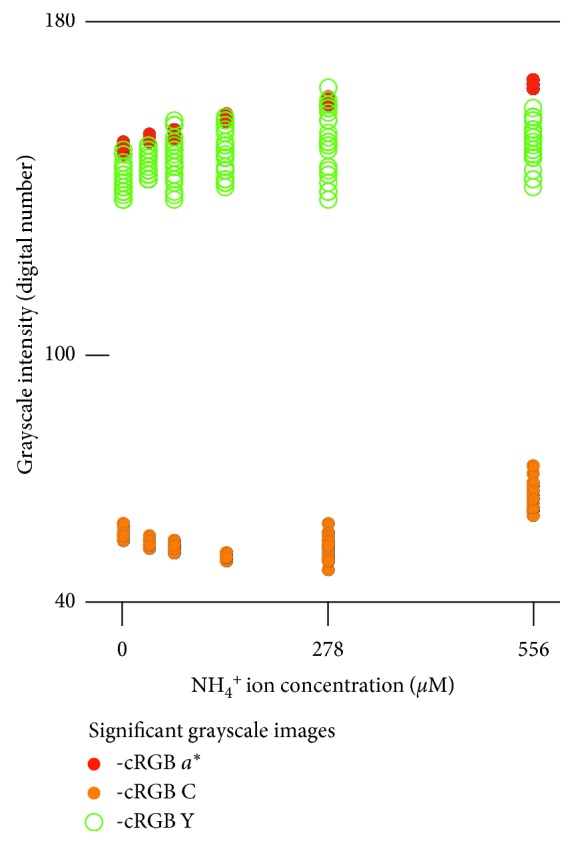
Relationships between ammonium ion concentration and the grayscale intensity values for the selected pixels in the significant grayscale images that provided the best regression model for determination of ammonium ion concentration.

Quantification of NH_4_^+^ that relied on deep learning was less successful than that of chemical oxygen demand (Figure 6) although no signals of overtraining were observed. For both the ten and 83 images' datasets, the normalized mean absolute error and *R*^2^ were around 3% and 0.99, respectively. Regression coefficients were 0.953 (10 images) and 0.954 (83 images). These values deviated from 1.0, which was considered to be a perfect match, shown as red oblique lines in Figure 6. The most critical part was that the pack containing no NH_4_^+^ was shown to contain 35 *µ*M NH_4_^+^. This indicates the possibility of a false positive result if users rely on deep learning and the other techniques that were applied in this study. Here, multiple regression markedly improved the precision, resulting in a smaller normalized mean absolute error of 2.34%. Overall, the regression was very accurate, with a coefficient of 1.001 and a constant of 0.000. The multiple regression model was as follows:

(3) $$\begin{array}{c} {{\text{NH}}_{4}}^{+}\text{ concentration}\left(µ\text{M}\right)=0.195\times ‐\text{cRGB }a^{*}+0.0798\times ‐\text{cRGB C}+0.0569\times ‐\text{cRGB Y}-30.9, \end{array}$$

where ‐cRGB *a*^*∗*^ and the other names of the grayscale images indicated the grayscale intensity values for the selected training pixels in the grayscale images. The variance inflation factor was 2.50 (‐cRGB *a*^*∗*^) or less, indicating good reliability for the three coefficients. The grayscale intensity for the ‐cRGB *a*^*∗*^ images had a more significant linear correlation with the NH_4_^+^ concentration (*R*^2^ = 0.916) compared with the others for which the *R*^2^ values were smaller than 0.460 (Figure 7). Although the *R*^2^ value of 0.916 for the ‐cRGB *a*^*∗*^ grayscale intensity may seem to be large, it was much smaller than the 0.992 result for the multiple regression model (Figure 6). This indicates that the multiple regression model was achieved using the combination of less linear variables as previously described [4].

**Figure 8:**
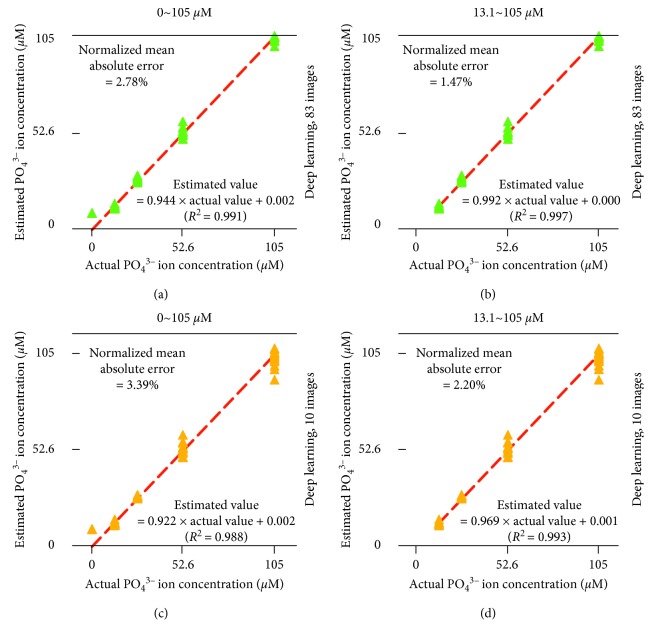
Estimation of phosphate ion (PO_4_^3−^) concentration by using deep learning based on the 10 and 83 grayscale images derived from the coloration image (Figure 3). The oblique red lines indicate perfect matching between actual and estimated values.

In quantification of PO_4_^3−^, no signals of overtraining were recognized. However, the quantification had a similar problem to that of NH_4_^+^. The pack containing no PO_4_^3−^ was indicated as containing a small but detectable amount of PO_4_^3−^ (Figure 8). The normalized mean absolute error and *R*^2^ were comparable to those for the NH_4_^+^ determination (Figure 6). A regression coefficient of 0.922 for deep learning with the original ten images' dataset was worse than that of 0.953 for NH_4_^+^ determination by deep learning with the original ten images' dataset (Figure 6). Unfortunately, multiple regression did not provide reliable results to improve the poor performance. However, these poor results could be circumvented by limiting the range of PO_4_^3−^ concentration to be determined. The deep learning-estimated values for 0 *µ*M PO_4_^3−^ were eliminated (Figure 8). Although the range of PO_4_^3−^ concentration that the method can determine became 13.1 to 105 *µ*M PO_4_^3−^, the error statistics were significantly improved.

The 83 images' dataset improved the accuracy of the NH_4_^+^ and PO_4_^3−^ determination (Figures 6 and 8). Principal component analysis of color profiles based on the 331 pixels in the Microsoft standard colors and gamut is provided in Table 1. The table shows a data structure based on the grayscale intensity values for the 331 pixels in the 83 grayscale images. Table 1 presents the results regarding significant principal components with eigenvalues of 1 or greater [23]. Principal component loading values for the 83 grayscale images are presented. In a previous study, adding the grayscale images was shown to provide more opportunities to obtain new patterns of changes in the grayscale intensity [4]. When a user of test strips relies on multiple regression, the different patterns revealed by the principal components were effective in determining the chemical characteristics by combining the best grayscale images that carry the best combination of patterns in the grayscale intensity [4]. In finding differences among color profiles of image pixels representing agricultural plots [24] or tropical forest canopies [25], the 14 grayscale images from the RGyB and RG-yB pseudocolor images together with the RGB yellow hybrid image (Figure 1) generated minor but significant principal components. In this study, among the 15 grayscale images, nine had the highest loadings on the first principal component (Table 1). Thus, the additional 58 grayscale images further diversified the data structure. The Microsoft standard colors and gamut are thought to include more colors than the images of the agricultural plots and the forest canopies. The greater number of colors is also thought to have revealed the more diversified data structure.

Additionally, most of the grayscale images from the RmGB and R-mGB pseudocolor images had the heaviest loadings on the second or third principal component while none of them had the greatest loadings on the first principal component. This was fortunate because the grayscale images even more significantly added the unique information to the original ten and 15 grayscale images examined in previous studies [24, 25]. The second and third principal components were comparable in importance compared with the first principal component, and many grayscale images derived from the RmGB and R-mGB pseudocolor images formed significant regression models in another study [4]. Additionally, the grayscale images derived from the cRGB and -cRGB pseudocolor images had the greatest loadings on the first or third principal component (Table 1). These unique loading patterns indicate greater dimensionality of changes in pixel color in the Microsoft standard colors and the gamut revealed using the pseudocolor imaging method. RGB cyan, magenta, and yellow hybrid images were likely to broaden the dimensionality because these hybrid images had uniquely different loading patterns on the most significant three principal components (Table 1).

Because of the high feasibility, PackTest products can be used to quantify the chemical characteristics of various liquid samples using optical scanners and other digital imaging devices such as digital cameras and colorimeters [26] combined with deep learning and pseudocolor imaging. The combination is applicable to solid samples and similar coloration-based tools including test strips. Examples of possible applications are eliminating difficulties in the positive or negative judgment of urinary creatinine [27], improving somewhat inaccurate description of plant biomass growth [28], and confirming the quality of foods [29] and various other materials. The application may be extended to analyses of samples in various places including laboratories, hospitals [30], schools [31], and homes [7], where the color reading-based tools can be easily introduced.

## 4. Conclusions

Accurate and highly feasible quantification of chemical characteristics of the liquid sample is possible using PackTest products. The SONY neural network console, as a free-of-charge deep learning tool, was the first choice for processing the grayscale intensity values derived from color readings of the pack that showed a color as a result of the chemical reaction. Multiple regression revealed the possibility to be a substitute for deep learning when deep learning resulted in unsatisfactory accuracy. Another solution was to limit the range of levels/concentrations of the chemical characteristics to be measured. These techniques to maximize the quantification accuracy are supported by enhanced information through pseudocolor imaging processes.

## Figures and Tables

**Table 1:** Loadings on principal components (eigenvalue > 1) for the grayscale images to show the structure of the grayscale intensity data for the 331 pixels representing the Microsoft Office standard colors and gamut in the 83 grayscale images.

(Pseudo)color image as the source of grayscale images	Grayscale image	Principal components (explaining percentage, eigenvalue)						
		1 (30%, 25)	2 (28%, 23)	3 (24%, 20)	4 (5%, 4)	5 (3%, 3)	6 (2%, 2)	7 (2%, 2)
RGB true color image	R	0.396	0.856	−0.158	0.179	−0.080	−0.039	0.161
	G	0.863	−0.446	0.115	0.072	0.053	−0.131	−0.028
	B	−0.055	0.051	0.949	0.115	−0.204	−0.051	−0.120
	C	0.483	0.761	−0.279	0.276	−0.016	−0.003	−0.061
	M	0.761	−0.608	0.084	0.090	−0.005	−0.108	−0.006
	Y	−0.312	0.162	0.857	0.124	−0.264	−0.080	−0.047
	K	0.528	0.301	0.539	−0.341	0.207	0.342	0.035
	*L* ^*∗*^	0.968	−0.030	0.165	0.124	0.015	0.077	−0.067
	*a* ^*∗*^	−0.510	0.831	0.089	0.087	−0.008	0.132	0.081
	*b* ^*∗*^	0.600	−0.023	−0.779	−0.016	0.157	0.044	−0.011
	RGB cyan	0.504	−0.256	0.774	−0.029	−0.089	0.037	0.222
	RGB magenta	0.233	0.706	0.598	0.127	0.221	−0.043	−0.088
	RGB yellow	0.876	0.395	−0.071	−0.063	−0.144	0.093	−0.168

RGyB	C	0.675	0.619	−0.123	0.313	−0.044	−0.007	0.133
	M	0.783	−0.557	0.122	0.147	0.083	0.007	−0.076
	Y	0.574	0.668	−0.105	−0.266	−0.217	−0.218	−0.013
	K	0.656	0.356	0.017	−0.485	−0.187	−0.060	−0.241
	*L* ^*∗*^	0.983	0.021	0.021	0.079	−0.041	0.041	−0.118
	*a* ^*∗*^	−0.301	0.913	−0.195	−0.002	−0.127	0.101	0.064
	*b* ^*∗*^	0.210	−0.804	0.185	0.388	0.242	−0.088	0.137
RG-yB	C	0.450	0.834	−0.165	0.160	−0.116	0.053	0.065
	M	0.770	−0.569	0.142	0.028	0.065	−0.157	−0.034
	Y	−0.845	−0.274	0.072	0.139	0.151	−0.056	0.181
	K	0.252	−0.110	−0.092	0.585	0.138	0.563	−0.081
	*L* ^*∗*^	0.956	−0.112	0.030	0.235	0.015	0.046	−0.059
	*a* ^*∗*^	−0.605	0.718	−0.181	0.186	−0.032	0.119	0.165
	*b* ^*∗*^	0.926	0.284	−0.047	−0.048	−0.120	0.077	−0.165

RmGB	C	0.367	0.811	−0.231	0.266	−0.151	−0.010	0.193
	M	0.361	0.811	−0.295	0.223	−0.087	−0.040	0.196
	Y	0.210	−0.804	0.185	0.388	0.242	−0.088	0.137
	K	0.235	0.616	0.553	−0.120	0.359	−0.147	−0.103
	*L* ^*∗*^	0.392	0.883	−0.025	0.197	−0.007	−0.034	0.121
	*a* ^*∗*^	−0.288	−0.488	0.696	0.028	0.234	0.125	−0.278
	*b* ^*∗*^	0.303	0.542	−0.747	0.035	0.041	−0.020	0.212

R-mGB	C	0.323	0.773	−0.250	0.290	0.212	0.150	0.085
	M	−0.358	−0.862	0.179	−0.187	0.028	0.034	−0.168
	Y	0.002	0.292	0.846	0.135	−0.320	−0.106	−0.032
	K	−0.101	−0.055	0.358	0.422	−0.145	0.488	−0.176
	*L* ^*∗*^	−0.322	−0.731	0.244	0.196	0.081	0.348	−0.214
	*a* ^*∗*^	0.373	0.877	−0.014	0.235	−0.072	−0.023	0.136
	*b* ^*∗*^	−0.046	−0.295	−0.842	−0.069	0.344	0.195	0.014

cRGB	C	0.638	−0.355	0.629	0.022	−0.077	−0.112	0.056
	M	0.837	−0.464	−0.052	0.181	0.095	−0.059	−0.038
	Y	−0.315	0.197	0.841	0.112	−0.226	−0.035	−0.187
	K	0.424	−0.150	0.685	−0.272	−0.091	0.034	0.377
	*L* ^*∗*^	0.763	−0.386	0.487	0.043	−0.017	−0.019	0.132
	*a* ^*∗*^	−0.619	0.331	0.640	−0.108	−0.169	0.135	0.147
	*b* ^*∗*^	0.656	−0.359	−0.601	−0.073	0.188	−0.022	0.153

-cRGB	C	−0.145	0.066	−0.721	0.349	0.125	−0.060	−0.466
	M	0.847	−0.444	0.145	0.007	0.052	−0.126	0.047
	Y	−0.297	0.196	0.875	0.014	−0.223	−0.014	−0.066
	K	0.200	−0.182	0.440	0.331	−0.070	0.309	−0.004
	*L* ^*∗*^	0.809	−0.432	−0.039	0.281	0.083	−0.082	−0.184
	*a* ^*∗*^	−0.855	0.436	−0.070	0.083	−0.061	0.140	−0.104
	*b* ^*∗*^	0.423	−0.242	−0.837	0.054	0.207	−0.048	−0.068

RmGyB	C	0.405	0.855	0.006	0.309	0.021	0.005	0.052
	M	0.095	0.630	0.648	0.058	0.236	−0.184	−0.109
	Y	0.826	0.241	−0.394	−0.018	−0.235	0.092	−0.109
	K	0.472	0.357	0.420	−0.561	0.261	0.180	−0.008
	*L* ^*∗*^	0.349	0.761	0.490	0.107	0.193	0.012	−0.053
	*a* ^*∗*^	0.462	0.049	−0.767	0.020	−0.305	0.213	0.152
	*b* ^*∗*^	−0.599	0.394	0.546	0.217	0.315	−0.143	0.096

cRGyB	C	0.449	−0.427	0.735	0.037	0.005	−0.025	0.217
	M	0.888	−0.404	−0.003	0.194	0.003	−0.029	−0.056
	Y	0.676	0.452	−0.126	−0.306	−0.303	−0.196	−0.206
	K	0.514	0.343	0.397	−0.569	0.201	0.174	−0.023
	*L* ^*∗*^	0.856	−0.315	0.394	0.038	0.018	0.018	0.059
	*a* ^*∗*^	−0.599	0.626	0.352	−0.172	−0.070	0.245	0.051
	*b* ^*∗*^	−0.023	−0.783	0.505	0.139	0.161	−0.088	0.261

cRmGB	C	0.485	−0.315	0.754	0.063	−0.134	0.142	0.143
	M	0.052	0.805	0.433	0.140	0.233	−0.245	−0.078
	Y	−0.101	−0.103	0.918	0.219	−0.196	−0.025	−0.151
	K	0.454	0.391	0.491	−0.472	0.298	0.143	−0.008
	*L* ^*∗*^	0.377	0.534	0.723	0.050	0.168	0.069	−0.044
	*a* ^*∗*^	0.203	−0.855	0.257	−0.099	−0.285	0.127	0.191
	*b* ^*∗*^	0.405	0.307	−0.722	−0.160	0.334	0.116	0.167
cRmGyB	C	0.534	−0.094	0.802	0.101	−0.029	0.045	0.108
	M	0.065	0.783	0.505	0.145	0.241	−0.144	−0.082
	Y	0.889	0.133	−0.254	−0.057	−0.267	0.035	−0.124
	K	0.468	0.335	0.407	−0.576	0.253	0.200	−0.002
	*L* ^*∗*^	0.434	0.548	0.676	0.034	0.170	0.088	−0.041
	*a* ^*∗*^	0.400	−0.817	−0.039	−0.160	−0.264	0.165	0.177
	*b* ^*∗*^	−0.593	0.073	0.718	0.135	0.279	−0.071	0.121

## Data Availability

The grayscale intensity data used to support the findings of this study are available from the corresponding author upon request.
